# Post-COVID breathlessness: a mathematical model of respiratory processing in the brain

**DOI:** 10.1007/s00406-023-01739-y

**Published:** 2024-03-19

**Authors:** Dina von Werder, Franziska Regnath, Daniel Schäfer, Rudolf Jörres, Nadine Lehnen, Stefan Glasauer

**Affiliations:** 1 Institute of Medical Technology, Brandenburg University of Technology Cottbus-Senftenberg, Lipezker Strasse 47, 03048 Cottbus, Germany; 2 Graduate School of Systemic Neurosciences, Ludwig-Maximilians-Universität München, Munich, Germany; 3 Klinikum rechts der Isar, Department of Psychosomatic Medicine and Psychotherapy, Technical University Munich, Munich, Germany; 4 TUM Graduate School, Faculty of Sport and Health Sciences, Technical University Munich, Munich, Germany; 5 Institute and Outpatient Clinic for Occupational, Social and Environmental Medicine, University Hospital, LMU, Munich, Germany; 6 Comprehensive Pneumology Center Munich (CPC-M), Member of the German Center for Lung Research (DZL), Munich, Germany; 7 Faculty of Health Sciences Brandenburg, Brandenburg University of Technology Cottbus-Senftenberg, Cottbus, Germany

**Keywords:** Post-COVID, Breathlessness, Bayesian brain, Respiratory processing, Predictive processing

## Abstract

Breathlessness is among the most common post-COVID symptoms. In a considerable number of patients, severe breathlessness cannot be explained by peripheral organ impairment. Recent concepts have described how such persistent breathlessness could arise from dysfunctional processing of respiratory information in the brain. In this paper, we present a first quantitative and testable mathematical model of how processing of respiratory-related signals could lead to breathlessness perception. The model is based on recent theories that the brain holds an adaptive and dynamic internal representation of a respiratory state that is based on previous experiences and comprises gas exchange between environment, lung and tissue cells. Perceived breathlessness reflects the brain’s estimate of this respiratory state signaling a potentially hazardous disequilibrium in gas exchange. The internal respiratory state evolves from the respiratory state of the last breath, is updated by a sensory measurement of CO_2_ concentration, and is dependent on the current activity context. To evaluate our model and thus test the assumed mechanism, we used data from an ongoing rebreathing experiment investigating breathlessness in patients with post-COVID without peripheral organ dysfunction (*N* = 5) and healthy control participants without complaints after COVID-19 (*N* = 5). Although the observed breathlessness patterns varied extensively between individual participants in the rebreathing experiment, our model shows good performance in replicating these individual, heterogeneous time courses. The model assumes the same underlying processes in the central nervous system in all individuals, i.e., also between patients and healthy control participants, and we hypothesize that differences in breathlessness are explained by different weighting and thus influence of these processes on the final percept. Our model could thus be applied in future studies to provide insight into where in the processing cascade of respiratory signals a deficit is located that leads to (post-COVID) breathlessness. A potential clinical application could be, e.g., the monitoring of effects of pulmonary rehabilitation on respiratory processing in the brain to improve the therapeutic strategies.

## Introduction

### Post-COVID breathlessness

Persistent breathlessness is estimated to affect more than 25% of patients after COVID-19 [1]. While some patients present with impaired lung function and carbon monoxide diffusing capacity [2], others have neither measurable pulmonary [3, 4] nor cardiac impairments [5] despite profound breathlessness. In general, there is only a moderately strong relationship between peripheral organ dysfunction and patients’ breathlessness, and a considerable number of patients lack any measurable organic symptom correlate [1]. Recently, concepts based on the processing of respiratory information in the brain have been developed that describe how persistent breathlessness that is not sufficiently explained by organ dysfunction could manifest [6–10]. These concepts highlight that perception of symptoms occurs in the brain, even if the initial cause resides in body periphery, and that symptoms can be just as authentic and disabling when peripheral organs are intact, but information relayed from sensors to the brain is misprocessed. Therefore, investigating how bodily signals are processed in the brain should be an integral part of the search for possible disease mechanisms in addition to the examination of peripheral organ impairments.

### A new perspective on breathlessness

The environment around us is constantly changing. To keep the body in homeostasis, the brain must monitor all relevant processes in the body and adjust them as soon as they exceed setpoints such as a certain core temperature, pH or glucose level [11]. In the case of breathing, different receptors signal information about lung mechanics, cardiac function, carbon dioxide (CO_2_) concentration and pH levels in the blood. The brain needs to measure and integrate these signals to obtain information about the current respiratory state, i.e., the gas exchange between the environment, lungs, and tissue cells [12, 13]. This involves two problems: (1) Sensory information coming from receptors is inherently noisy which makes sensing of a signal prone to errors. (2) Sensory input always follows an event and consequently is delayed. Therefore, reactive control of bodily states will often be too slow leading to over- or undershooting the desired setpoint, which could, e.g., in the case of pH levels, be life-threatening.

**Fig. 1:**
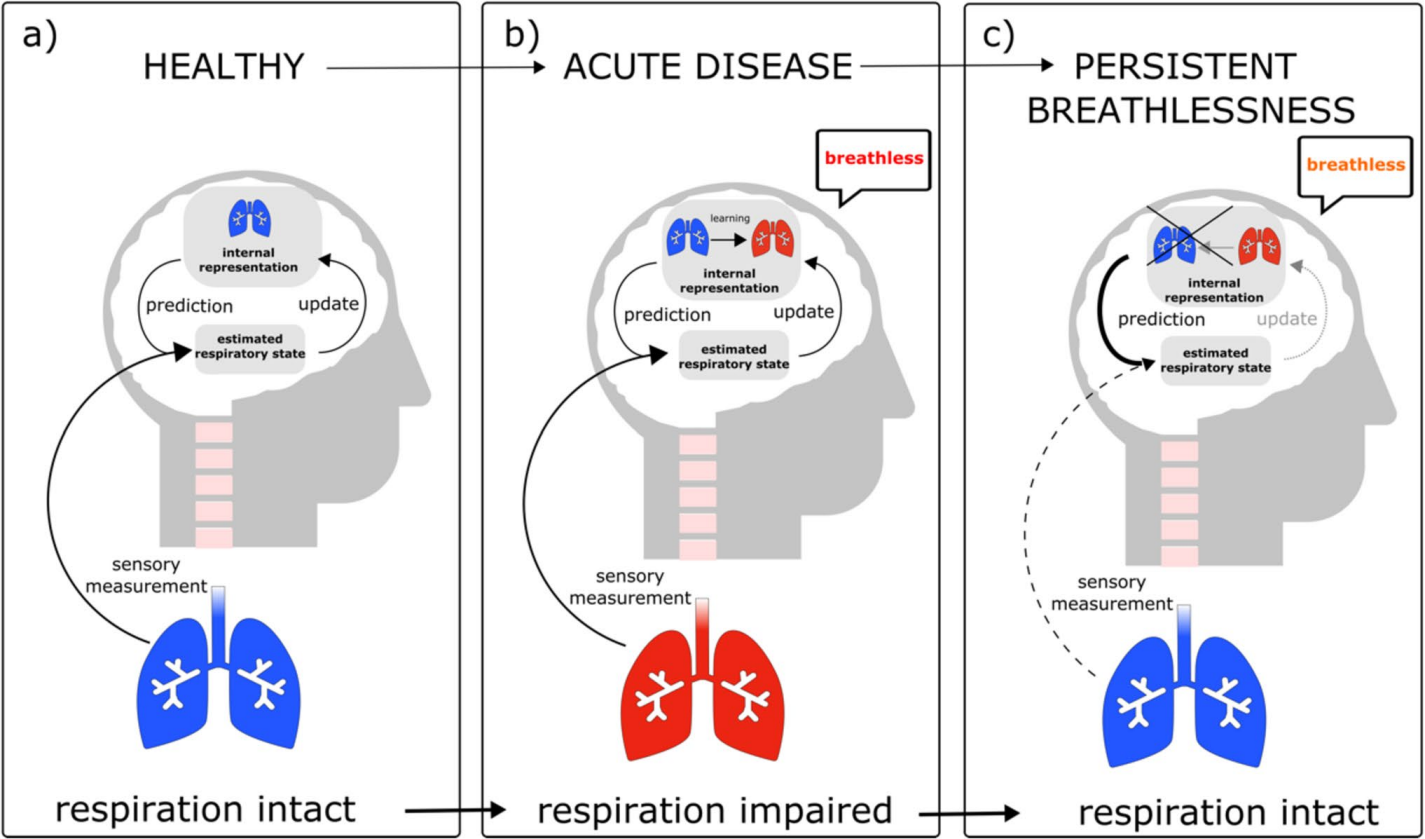
Development of breathlessness perception. **a**–**c** The brain holds an internal representation how bodily states evolve over time. Based on this, it can inform predictions about sensory input and use these predictions to optimally estimate the actual sensory input in a noisy environment. The brain’s best estimate is thus always a combination between prediction and sensory measurement. Each component can be weighted differently, according to how precise it is (Bayes law). **b** During acute disease, respiration can be impaired, and the internal representation is adapted to this diseased state. **c** When the lung recovers and respiration is intact, but the internal representation not updated, predictions are developed based on an internal representation that still assumes impaired respiration. If sensory input is noisy (dashed line) and predictions assumed to be very precise (thick line), predictions will be weighted more strongly in the estimation process of the respiratory state. Thus, even though sensory input signals intact respiration, inadequate predictions of diseased respiration can bias the estimate toward a respiratory state signaling impaired gas exchange. This can subsequently lead to breathlessness in the absence of any sensory input signaling impaired respiration

This implies that in many scenarios, reactive control mechanisms will not be sufficient. Conversely, it is crucial that the brain predicts deviations from setpoints in advance and adjusts breathing in anticipation of actual changes, e.g., in pH. To predict future changes of bodily states, the brain needs to form an internal representation that describes how such bodily states evolve over time (see Fig. 1). This internal representation is often called an internal model. It needs to be dynamically adapted based on newly available information. For example, changes such as increased lung ventilation due to training, or decreased lung function due to disease (as in Fig. 1b) need to be incorporated. This means that the internal representation is built from past experiences. Based on these, predictions can be developed (see Fig. 1) to handle noisy measurements and obtain an optimal estimate of the underlying body state [9, 14, 15]. This is comparable to driving on a familiar road at night: even if visibility is poor, our knowledge of how the road is developing improves our perception and makes driving easier. In a similar way, the brain obtains an optimal estimate of the actual underlying body state from the combination of sensory input and prediction. The relative contributions of noisy measurements and predictions (see Fig. 1c) are determined by their relative precision. If sensory input is very noisy and imprecise (like when driving at night and vision is poor), more reliance will be put on predictions (our knowledge of the road), and the resulting estimate is shifted toward these. Thus, predictions will dominate the estimate of the body state (see Fig. 1c). In contrast, if sensory input is precise (driving during the day and good vision), the brain’s estimate of the body state will more closely reflect the actual sensory input. The brain’s best educated estimate about the underlying body state is thus a combination of predictions based on internal representations and sensory input. This is described by Bayes’ Law, a statistical framework that can explain different perceptual phenomena [16, 17] and is often used to model perception [15]. It is important to highlight that the brain’s estimate about body states is not necessarily consciously perceivable and that probably a further step is necessary that translates this estimate into conscious perception.

While internal representations are crucial to correctly interpret the noisy information around us and to deal with bodily perturbations in an adequate and timely manner, visual illusions demonstrate that predictions based on internal representations can also misdirect perception. Visual illusions (such as the checkerboard illusion [18]) are often caused by strong predictions that bias our perception, leading to a discordance between the perceived reality and the physical reality. Similar to perception of stimuli arising *outside* the body, internal representations can bias perception of stimuli arising *inside* the body [19]. If the internal representation about the processes causing the respiratory state is defect, measurements will be incorrectly interpreted, and breathlessness could arise even if the sensory input does not signal any abnormalities—just as an optical illusion is perceived and becomes one’s own reality despite not corresponding to physical reality. Importantly, even though objective knowledge about the actual physical reality is present, it usually does not ‘correct’ perception.

At present it is unclear how and where these internal representations are implemented in the brain, although there is some evidence that the insula [20, 21] and cerebellum [22–24] are involved in updating and maintaining internal representations. Here, mathematical models can provide relevant insights by revealing constraints to which the physiological mechanisms must be subjected. Such models implement a quantitative description of assumed internal representations and estimation processes of bodily states. In our model, we assume an internal respiratory state that describes gas exchange between environment, lung and tissue cells. The current internal representation evolves from that of the last breath via updating from sensory measurements of CO_2_ concentration in the blood and cerebrospinal fluid. The perceived breathlessness reflects the brain’s estimate of this respiratory state signaling a normal versus potentially dangerous disequilibrium in gas exchange.

In the present work, we test the plausibility of this hypothesized mechanism by evaluating whether our model can describe the relationships between individual breathlessness ratings and CO_2_ levels measured in a rebreathing experiment.

By writing down our proposed mechanism as a quantitative mathematical model, we render our theory about processing of respiratory information in the brain testable. We hypothesize that breathlessness ratings from a very heterogeneous sample including healthy participants and patients with post-COVID can be simulated by a model that assumes the same underlying processes in all individuals and that differences in breathlessness are explained by different weighting and thus influence of these processes on the final percept.

## Methods

The current study is part of the innovative training network ETUDE (Encompassing Training in fUnctional Disorders across Europe; https://etude-itn.eu/), ultimately aiming to improve the understanding of mechanisms, diagnosis, treatment and stigmatization of Functional Disorders [25].

### Experimental paradigm

Experimental data were acquired using an experimental paradigm that is a variation of Read’s rebreathing method [26] and was previously used to investigate, e.g., medically unexplained breathlessness [27], as well as chronic fatigue and fibromyalgia [28]. Participants breathed through a mouthpiece that was connected to a Y-valve behind a visual barrier. The experimenter was located behind the barrier and could let the participant breathe either room air or air from a rebreathing bag. The rebreathing bag was initially filled with a gas mixture of 5% CO_2_ and 95% O_2_ (Carbogen, *Linde*). Due to rebreathing from this closed system, the inhaled CO_2_ concentration gradually increased leading to hypercapnia and breathlessness.

During the experiment, we recorded CO_2_ concentration in breathed air (capnograph, *Hans Rudolph*), peripheral oxygen saturation (pulse oximetry, *Nonin Xpod*) and respiratory flow rate (pneumotachograph, *Hans Rudolph*) with a sampling rate of 50Hz. For this study, we calculated single breath data for CO_2_ concentration. End-tidal CO_2_ (etCO_2_) was obtained by taking the maximum CO_2_ concentration exhaled in each breath. These single breath data were averaged over 10s intervals. Participants were instructed to rate their breathlessness on a scale from 0 (not at all) to 100 (unbearable) every 10s when an auditory cue was presented. They were informed that they would breathe air with different concentrations of CO_2_ and O_2_ that can induce either a feeling of breathlessness or no symptoms at all. However, at no point in the experiment, the actual source of breathed air was known to them. The experiment started with a baseline phase, during which participants inhaled room air for 60s. This was followed by a rebreathing phase for 150s and a subsequent recovery phase with room air for another 150s.

### Participants

We recruited patients at specialized post-COVID clinics in university hospital settings who presented with post-COVID breathlessness not explained by peripheral cardiorespiratory or neurological impairments. All patients needed to provide a PCR test documenting the initial SARS-CoV-2 infection and had to be suffering from post-COVID symptoms for at least 3 months. Data collection for this rebreathing study is still ongoing, but we consider it worthwhile to inform other researchers on our modeling approach using first results. For evaluating the model, we included data from the first 5 patients (mean age ± standard deviation: 34.2 ± 13.7 years, 4 female). Healthy control participants were recruited through the intranet of the Klinikum rechts der Isar, Technical University Munich, as well as through advertisement (flyers) outside of the clinic. For this study, we included 5 healthy controls participants (mean age ± standard deviation: 35.0 ± 15.5 years, 4 female) who were matched by age and gender to the 5 patients.

On the day of the experiment, lung function tests (spirometry and diffusing capacity for CO) and a standardized neurological examination were performed to rule out any organ impairment on that very day. None of the included participants nor patients showed signs of impairment in these exams. In addition, we clinically characterized all participants using the gold standard for making DSM-5 diagnoses, i.e., the Structured Clinical Interview for DSM-5 disorders (SCID-5-CV). Furthermore, we used the patient health questionnaire (PHQ-15), a well-established tool which asks about the presence and severity of common bodily symptoms [29], and asked participants about the presence and severity of breathlessness in everyday life situations.

The study was designed in line with the Declaration of Helsinki, and the Ethics Committee of the Technical University Munich approved the study protocol prior to conduction. Informed consent was obtained from all individual participants included in the study.

### Model description

The brain is not passively waiting and then reacting to sensory input but rather actively predicting sensory input based on its internal representation how certain body states are generated. Accordingly, our main assumption for mathematical modeling is that the brain holds an internal representation of how bodily states related to breathlessness are changing over time and how these changes are linked to sensorily measurable quantities such as CO_2_ concentration. In the following, we will refer to the bodily state reflecting the gas exchange between environment, lung and tissue as “internal respiratory state”. We assume that perception of breathlessness reflects potentially dangerous levels of this state, like perception of pain reflects damage to the body. Perception of breathlessness thus represents the brain’s estimate of a respiratory state indicating disequilibrium in gas exchange that may cause dangerous pH levels in the blood.

**Fig. 2:**
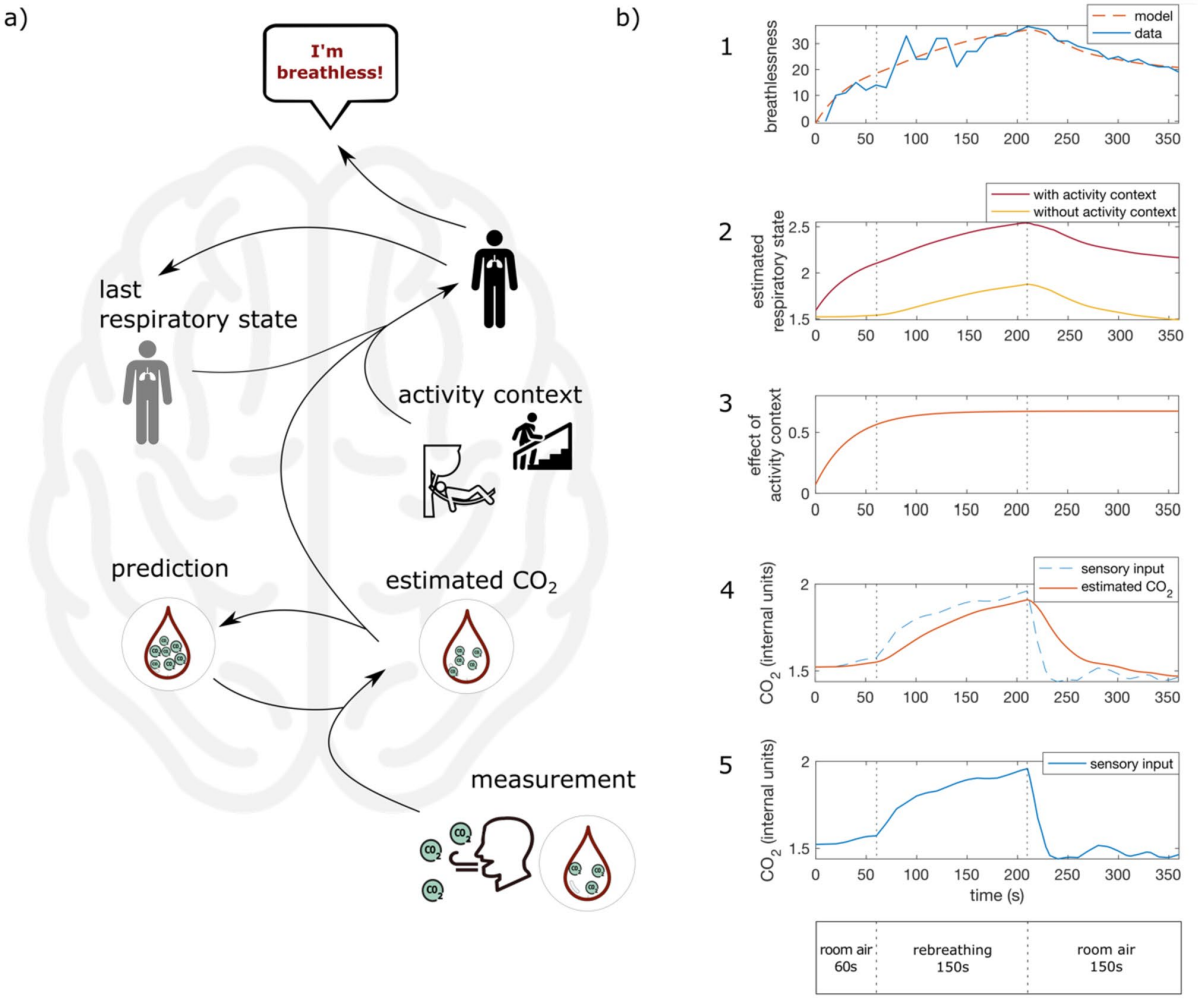
Model of breathlessness perception (**a**) and a visualization of the different processing steps (**b**). Measurement of CO_2_ concentration in the blood and cerebrospinal fluid (bottom, b5) is noisy and error-prone and thus needs to be combined with a prediction to obtain an estimate of the actual underlying CO_2_ concentration (orange, solid line in b4). Note that this internal estimate can be different from the actual CO_2_ concentration and will be used to update predictions about future measurements. Furthermore, the current activity context plays a role (b3). Walking up a flight of stairs leads to a high activity context, which will increase the respiratory state, while resting evokes a low activity context and a lower respiratory state. Note that while the activity context is constant throughout the simulation, its effect (shown in b3) increases and saturates after about 2 min for this participant. The respiratory state describes the current gas exchange between environment, lung and tissue cells and is not consciously accessible. The respiratory state in the last breath is used to predict the current respiratory state and can be updated by the estimated CO_2_ concentration as well as the activity state. How much the estimated CO_2_ concentration is taken into account can vary. If the sensory update is taken into account only to a very small extent, the respiratory state is mainly influenced by the prediction based on the last respiratory state and the current activity context. Thus, even though sensory measurements signal an improvement in CO_2_ levels (b5, in last phase with room air), the respiratory state signaling imbalances in gas exchange may show minor improvement (b2, in last phase with room air). Finally, the respiratory state needs to be translated into the perception of breathlessness (b1). Breathlessness thus reflects an internal respiratory state that signals a potentially dangerous imbalance in gas exchange

To construct our mathematical model of breathlessness perception (see Fig. 2, for the equations see Appendix), we first formulated a hypothesis about the brain’s internal representation how the respiratory state will evolve. This internal representation can then be used to form predictions to optimally estimate the internal respiratory state that is not directly accessible to the brain. All our following assumptions for the construction of the model are physiologically informed. For simplification, we assume that the respiratory state can be summarized in a single variable. We further assume that the state varies only slowly from one breath to the next and is influenced by the internal CO_2_ concentration as well as the current activity context. Walking up a flight of stairs would amount to a high activity context as compared to standing still. Similarly, our rebreathing paradigm can amount to a high activity context. The activity context thus describes the expected influence of an activity on respiratory demands. Importantly, it can be different between individuals. We chose exhaled CO_2_ concentration per breath as the sensory quantity to update the respiratory state since it is experimentally accessible and can be used to approximate arterial CO_2_ concentration [30] that is measured by chemoreceptors. Like the internal respiratory state, the exhaled CO_2_ concentration is assumed to vary only slowly from one breath to the next. Thus, we hypothesize that the current respiratory state evolves from the respiratory state in the last breath and is updated by the sensory CO_2_ state. This process describes the brain’s internal representation of how a respiratory state is generated.

For the estimation of the expected respiratory state, the brain needs to combine the measured CO_2_ concentration with the internal representation described above. Since measurement of the CO_2_ concentration is noisy and error-prone, the brain also needs to estimate the actual CO_2_ concentration. For this, the brain forms a prediction based on the internal representation that the CO_2_ level changes slowly, but randomly, from one breath to the next. This prediction can be combined with the measured CO_2_ concentration to optimally estimate the actual CO_2_ concentration. For this estimation process, the framework of Bayes law can be used. It shows that if sensory measurement is precise, the resulting CO_2_ estimate will primarily rely on the sensory measurement. However, if sensory uncertainty is high, the estimate will more closely reflect the prediction based on the internal representation. As Kalman Filters are generally applied to estimate states evolving over time from noisy measurements, we used this approach to formulate the Bayesian estimation process (for the equations see Appendix). The five free parameters of this estimator, which are considered to be characteristic for each individual, can be computed from the experimental CO_2_ and breathlessness data from each individual participant. They are (1) the ratio of measurement uncertainty and assumed random changes of CO_2_ concentration, (2) a weight factor describing how much the CO_2_ level influences the respiratory state in every breath, (3) a parameter for the assumed activity context, and (4,5) two scaling parameters for the transformation translating the respiratory state into breathlessness perception (formulated as linear transformation comprising an offset and a gain factor).

The resulting estimated breathlessness states from the estimation model were compared to the time course of the actual breathlessness ratings from participants in the experiment. The free parameters were fitted by minimizing least-squares between actual and estimated breathlessness rating using the in-built MATLAB function *lsqnonlin*.

### Model evaluation

To evaluate whether the observed breathlessness ratings could also be explained by a simpler model that assumes that breathlessness is a scaled and shifted version of sensory input, we compared our model to a linear regression model of the following form:

$$b = \beta_{0} + \beta_{1} * x + \varepsilon$$

with $b$: breathlessness, ${\beta }_{0}$_*:*_ intercept, ${\beta }_{1}$: regression slope, *x*: CO_2_ concentration measured in the experiment and $\varepsilon$: error term.

Furthermore, we tested whether simpler versions of our proposed model can explain breathlessness ratings equally well as the full version. Our proposed model describes the respiratory state as depending on the activity context, the respiratory state in the last breath and an estimate of the internal CO_2_ level. While sensory input (in this case internal CO_2_ level) will likely play a role to some extent in every participant, we kept this component but set up two new model variants where we (1) removed the activity context and (2) in another model removed the dependence on the respiratory state in the last breath.

Performance between the different model versions, i.e., (1) the full model, (2) without activity context and (3) without dependence on the last respiratory state and (4) the linear regression model was compared using Akaike Information Criterion (AIC) which evaluates the quality of a model fit while also taking into account the number of parameters and thus the risk of overfitting.

## Results

### Clinical characterization

Table 1 displays the clinical characteristics of all included patients and healthy control participants. These characteristics were not part of the modeling procedure, nor were they considered for statistical analyses to evaluate differences between patients and healthy control participants. Table 2 shows diagnoses of all participants as obtained with the clinical interview for DSM-5 disorders (SCID-5-CV).

**Table 1:** Clinical characterization of participants

	How breathless are you when…			PHQ-15 SCORE
	…at rest	…putting on clothes	…walking up the stairs one floor	
P1	2	5	7	6
P2	1	2	7	16
P3	1	3	8	21
P4	2	2	6	16
P5	0	3	5	17
H1	0	0	0	6
H2	0	0	0	2
H3	0	0	0	0
H4	0	0	1	6
H5	0	0	0	3

Left: Participants were asked how breathless they are in everyday situations. Breathlessness was rated on a scale from 0 (no breathlessness at all) to 9 (extreme breathlessness) in these different situations. Right: PHQ-15 scores of patients (P) and healthy controls (H). PHQ-15 scores of ≥ 5, ≥ 10, ≥ 15 represent mild, moderate and severe levels of somatization

**Table 2:** Diagnoses as obtained from SCID-5-CV interview of all participants. P - patient; H - healthy control participant

Participants	Diagnosis
P1	Major depressive disorder, single episode, unspecified
	Undifferentiated somatoform disorder
P2	Premenstrual dysphoric disorder
	Specific isolated phobias
	Undifferentiated somatoform disorder
P3	Major depressive disorder, single episode, moderate
	Generalized anxiety disorder
	Undifferentiated somatoform disorder
P4	Undifferentiated somatoform disorder
P5	–
H1	Bipolar disorder, in full remission
H2	Major depressive disorder, recurrent, in full remission
H3	–
H4	Specific isolated phobias
H5	–

### Modeling results

**Fig. 3:**
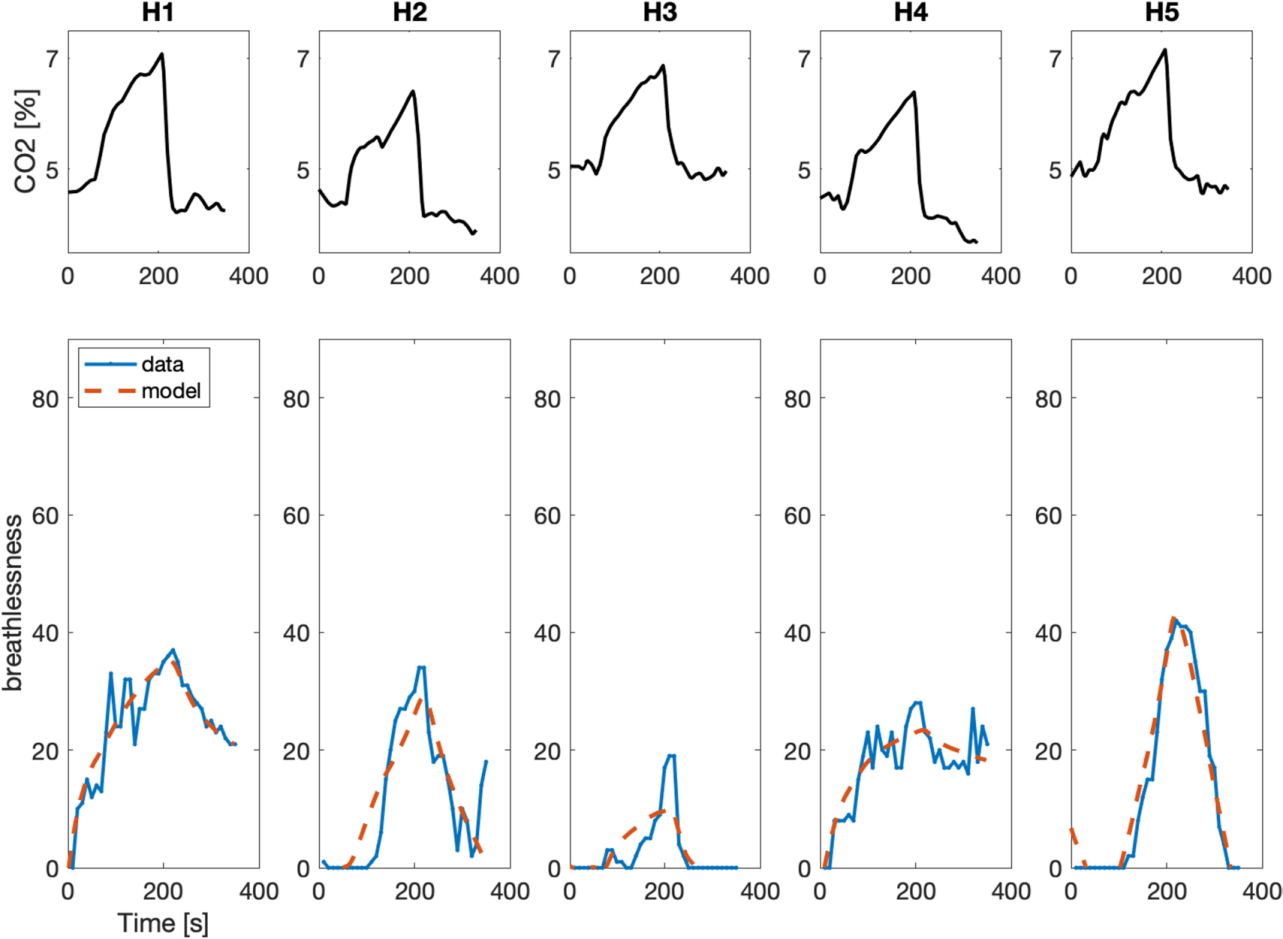
CO_2_ concentration in exhaled breath (**top**) and breathlessness ratings (blue) and model simulation (red dashed) (**bottom**) for individual, healthy control participants (H1: same data as in Fig. 2). Participants rated breathlessness on a visual analog scale from 0 to 100. H - healthy control participant

**Fig. 4:**
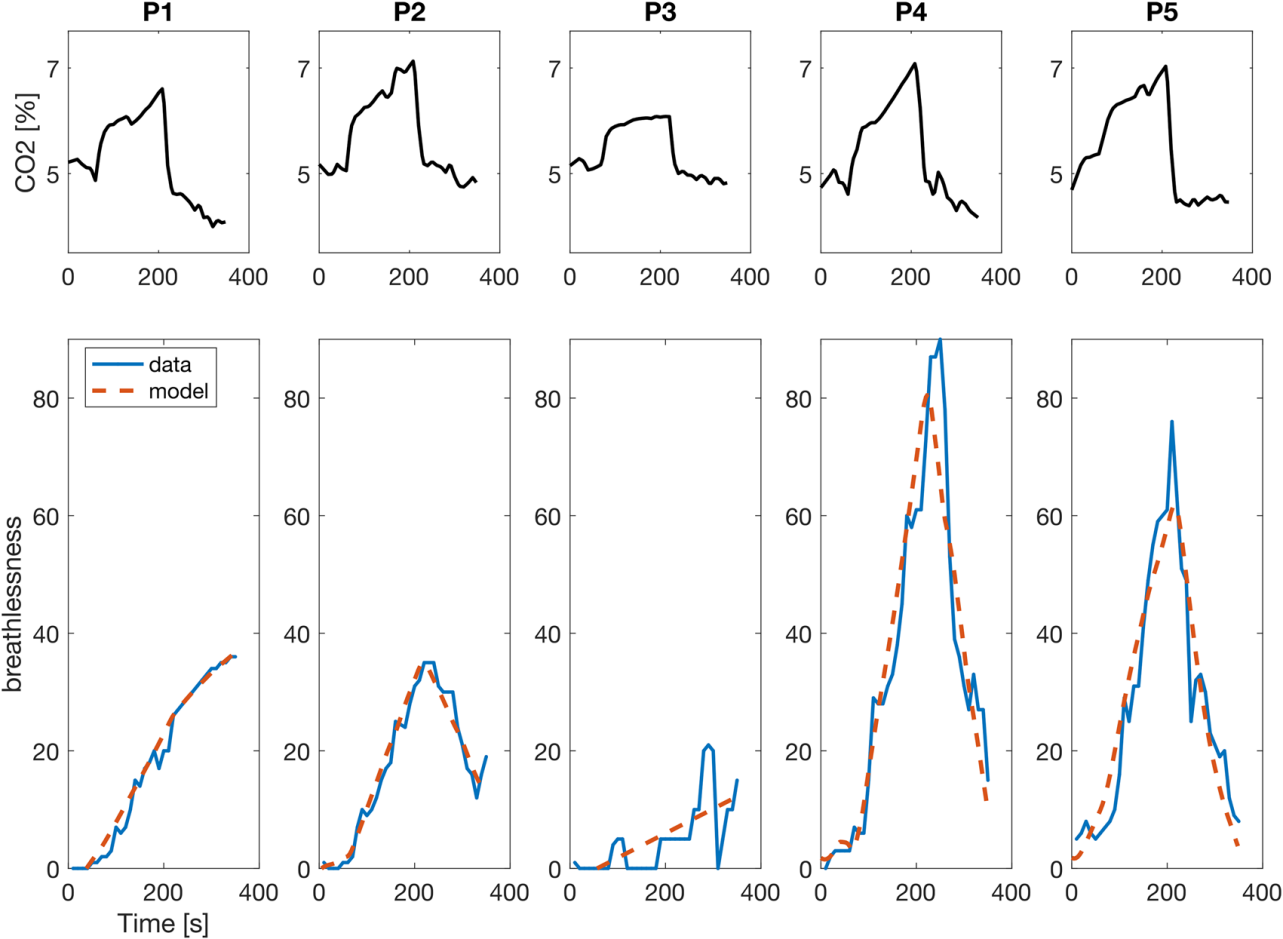
CO_2_ concentration in exhaled breath (**top**) and breathlessness ratings (blue) and model simulation (red dashed) (**bottom**) for individual patients with post-COVID breathlessness. Patients rated breathlessness on a visual analog scale from 0 to 100. P - patient

Although all participants inhaled air with the same CO_2_ concentrations, both at baseline and at the beginning of the rebreathing phase, i.e., received a similar sensory stimulus, breathlessness ratings varied considerably between participants (see Figs. 3 and 4). This was true both for the maximum perceived breathlessness and for the development of breathlessness over time. Differences in breathlessness patterns could be observed between the two groups; however, there were also substantial differences between individual patients, as well as between individual healthy participants. While some participants recovered rapidly after the rebreathing phase, i.e., breathlessness decreased back to low ratings, others remained breathless even when they were breathing room air (compare e.g., P1 to P5 in Fig. 4). Despite these very different patterns, our model showed good performance in its capability to replicate the observed time course of individual breathlessness. Using only CO_2_ concentration as input, it did not simply mirror this input but was also capable of describing breathlessness ratings that were uncoupled from the actual sensory input. This was for example the case in P1, where breathlessness increased throughout the experiment and stayed high, even though CO_2_ concentration had decreased back to baseline.

Table 3 shows Akaike Information Criterion (AIC) for variants of the proposed model as well as a linear regression model. The lower the AIC, the better the model performance. The diverse breathlessness patterns observed in the experiment were poorly explained by a linear regression model which assumes that breathlessness is a scaled and shifted version of the sensory input, i.e., CO_2_ concentration. In none of the participants, it performed better than the full proposed model or variants of it. Similarly, simpler version of our proposed model (1) without activity context and (2) without dependence on the respiratory state in the last breath predicted breathlessness ratings in general less well than the full model. A model variant without the activity context only led to slightly better predictions in 2 out of 10 participants. Similarly, the model variant without dependence on the respiratory state in the last breath only improved model prediction slightly in 2 out of 10 participants. However, in most participants, our full model showed a decisive improvement in model performance when compared to variants of it or the linear regression model.

**Table 3:**
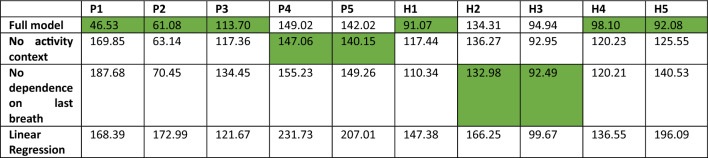
Akaike Information Criterion (AIC) for the full proposed model, variants with either no activity context or no dependence on the respiratory state in the last breath as well as a linear regression model

The lower the AIC, the better the model fit. Green: Lowest AIC, i.e., best model performance, for each participant. P - patient; H - healthy control participant

## Discussion

In this work, we provided a quantitative and testable model that describes how respiratory processing leads to breathlessness perception. According to our model, the brain needs to estimate a respiratory state by updating predictions based on the last respiratory state and an estimated CO_2_ concentration, while taking the current activity context into account. It showed good performance in describing highly heterogeneous time courses of individual breathlessness ratings obtained in our rebreathing experiment and outperformed other model variants as well as a linear regression model. Since the experimental data demonstrated very diverse breathlessness patterns, this might have required different mechanistic approaches for different subgroups. However, our model equipped with only one underlying mechanism was capable across all of these different, individual breathing patterns. Remarkably, it could also simulate breathlessness when it was uncoupled from the sensory CO_2_ stimulus (see P1, Fig. 4). It thereby provides a possible mechanism of how the same CO_2_ stimulus can be linked to different breathlessness patterns. Interestingly, only two patients with post-COVID (P4 and P5) developed strong breathlessness in the rebreathing phase. This shows that the patients in this study were not in general more sensitive to respiratory stimuli and thus experienced stronger breathlessness but that likely more complex dysfunctions in respiratory processing played a role that can result in more or less sensitive detection and response to these stimuli.

The parameter values of the model obtained from fitting the model output to experimental data describe how strongly each of the processes formulated in our model influence the final breathlessness percept. While the sample size in this study allowed to test whether the model in general can produce breathlessness ratings that are similar to experimentally obtained ratings, future studies with higher sample sizes are necessary to evaluate possible parameter differences between individuals as well as different groups. The parameters of the model provide specific insight into where in the processing of respiratory information a dysregulation might occur that leads to persistent breathlessness. For example, the internal CO_2_ state could be wrongly estimated. This could result from increased uncertainty of CO_2_ sensors, which leads to relying more on predictions than actually measured CO_2_ concentration. Then, the internal respiratory state would not reflect the actual underlying CO_2_ level. Another factor is the activity context, which, if wrongly estimated, might lead to increase of breathlessness even without changes in CO_2_ measurement. Our model thus allows to test within the same mechanism how different processes are weighted which could result in (post-COVID) breathlessness even though peripheral organ function is intact, and chemoreceptors signal a balanced gas exchange.

On a general level, the question remains how inadequate internal representations emerge. One possibility (see Fig. 1) could be that during the acute phase of COVID-19, the internal representation had to be adapted to a state of lung disease from viral infection (Fig. 1b). During this time, the adaptation was crucial to maintain homeostasis; however, it needs to be revised back to the healthy body state as soon as the infection resolves. If this does not take place (see Fig. 1c), sensory input signaling an intact lung would be interpreted with an internal representation referring to the diseased state, leading to symptom perception. A failure to update the internal representation could be due, for example, to persistent damage of respiratory chemoreceptive sensors or pathways. Persistent sensory changes in post-COVID have been reported for smell and taste, but also for other sensory inputs [31]. Such damage to respiratory chemoreception could also explain why breathlessness can be decoupled from actual CO_2_ level, as found in P1 (see Fig. 4). Furthermore, Sars-CoV-2-related changes in brain structure could play a role. In a longitudinal study comparing MRI scans before and after SARS-CoV-2 infection, Douaud et al. [32] found greater loss of gray matter and increased diffusivity, which is indicative of tissue damage in several brain regions, including the insula. Exploratory analyses have also shown loss of gray matter in the cerebellum. Both brain areas are involved in breathlessness perception and are assumed to store internal representations and to process prediction errors that arise when sensory input does not match predictions [33–35]. In addition, it is well known that stress [36] and mental health conditions such as anxiety [37, 38] interfere with how bodily signals are processed.

A discordance between symptoms and lung function parameters such as forced expiratory volume (FEV_1_) is a well-known phenomenon in respiratory diseases such as asthma [39–41]. However, symptoms decoupled from organ dysfunction are not specific to respiratory diseases but rather can be found in any field of clinical medicine [42]. Experimental approaches have been developed to test altered processing of body signals as a cause of these symptoms. For example, Lehnen et al. [43] developed an experiment that challenges the interaction between sensory input and internal model to study functional dizziness in patients with intact organ function which allowed to detect markers indicating dysfunctional sensorimotor processing [44, 45]. This was transdiagnostically extended to irritable bowel syndrome [46].

### Limitations

The fact that our mathematical model could simulate our experimental data does not necessarily mean that it is the only possible model. It is also still greatly simplified. For example, it is unlikely that CO_2_ concentration is the only sensory input used to update the respiratory state. Breathing also evokes, e.g., proprioceptive signals that provide information about lung mechanics such as the breathing frequency. In addition, for sudden changes in breathlessness perception that are decoupled from changes in CO_2_ concentration (see e.g., P3 & H3), our current model shows poor performance. Here threshold effects could be implemented in future versions of the model to allow simulations of such patterns. Furthermore, the sample size in this study only allowed to show that in general our model can predict different breathlessness patterns but did not allow for analysis of group differences, neither for model parameters nor for experimental data. Despite these limitations, we present our model at this stage of development because it could already describe experimental data very well, especially in view of the small set of parameters needed to describe a complex behavior.

### Outlook

Our model enables to test hypotheses about the processing of (post-COVID) breathlessness in the brain. While our hypothesis of how respiratory signals are processed in the brain is so far supported by results, further experimental tests are required to validate, and potentially refine it. Especially in post-COVID patients such as P1, an independent test of respiratory chemoreception could help to answer the question, whether sensory damage, e.g., to the carotid bodies or to central chemoreception [47], may have played a role in maintaining an inadequate internal representation of respiratory state. Another obvious consequence of the hypothesis would be that relief from breathlessness should be possible by readjusting the internal representation so that it adequately reflects a healthy state. One may assume that this already occurs during pulmonary rehabilitation programs, although not explicitly addressed [48]. Here, our model could provide a means to monitor which parameters are improved by rehabilitation. Finally, a possible method of providing improved sensory input is biofeedback, which has recently been suggested for post-COVID treatment of dysregulation of the autonomic system [49]. For example, monitoring the blood oxygenation level or, via transcutaneous CO_2_ monitoring, even the CO_2_ level, could show patients that their respiratory state is normal despite feeling breathless. Such a cognitive input might have a small effect but could help in gradually readjusting the internal representation.

## Data Availability

The data used in this study can be found on Open Science Framework (osf.io) via the following link: https://osf.io/srv5z/?view_only=325c37a979f74dc096ab189c9cdf772a.
